# Effect of adding indocyanine green to identifying sentinel lymph node in breast cancer patients after neoadjuvant chemotherapy

**DOI:** 10.1590/1806-9282.20241614

**Published:** 2026-01-09

**Authors:** Gizem Akçakoca, Yasin Tosun, Ecem Memişoğlu, Ömer Faruk İnanç, Cemal Hacıalioğlu, Kenan Çetin, Mehmet Velidedeoğlu, Hasan Fehmi Küçük

**Affiliations:** 1 Istanbul Kartal Dr. Lutfi Kırdar City Hospital, Department of General Surgery – Istanbul, Turkey.; 2 Istanbul University-Cerrahpaşa, Cerrahpaşa Faculty of Medicine, Department of General Surgery – Istanbul, Turkey.

**Keywords:** Breast cancer, Sentinel lymph node biopsy, Indocyanine green

## Abstract

**OBJECTIVE:**

The aim of the study was to examine the effectiveness of adding indocyanine green fluorescence to the dual method, (Radioisotope+Isosulfanblue) for sentinel lymph node biopsy after neoadjuvant chemotherapy (on sentinel lymph nodes identification).

**METHODS:**

The 101 patients included in the study were randomized equally into each group. Dual method containing indocyanine green (group 1) or dual method alone (group 2) was used for sentinel lymph node mapping.

**RESULTS:**

The sentinel lymph node identification rate was 86.9% in the dual method group and 94.6% in the triple method group. The detection rates of two or more sentinel lymph nodes were 82.6% in the dual group and 89.2% in the triple group. There was no statistically significant difference between both the groups (p>0.05).

**CONCLUSION:**

Indocyanine green-based sentinel lymph node biopsy detection will bring a significant change in the management of patients in centers where there is no access to a nuclear medicine department. Nevertheless, multicenter studies with larger patient samples are needed to confirm our results.

## INTRODUCTION

Axillary lymph node (ALN) status is one of the strongest prognostic factors in breast cancer patients, which guides the decision on adjuvant treatment. In patients who are clinically node-negative axilla (cN0), sentinel lymph node biopsy (SLNB) is an accepted approach for axillary staging^1^. Neoadjuvant chemotherapy (NAC) provides opportunities to facilitate breast-conserving surgery by causing tumor shrinkage, to ensure negativity of surgical margins, and to regulate the patient's adjuvant treatment by seeing the response to chemotherapy^2^. Additionally, 40–75% of patients show pathological complete response (pCR), including ALNs ^3,4-6^. SLNB can be performed to evaluate axillary nodal status in patients who are clinically negative after NAC. This procedure results in low morbidity^7,8^. However, previous studies have shown that SLNB after NAC has a false-negative rate (FNR) ranging from 8.4 to 4.2% and a lower sentinel lymph node (SLN) identification rate ranging from 80.1 to 92.9%. But, by presenting with T1-3/cN1 disease, finding three or more SLNs, and using a combined technique, FNR drops below 10%^2,9-11^. Today, the most commonly used methods for SLNB are radioisotope (RI) and Blue Dye. However, especially RI need for equipped facilities, and the need for an experienced nuclear medicine team. Although RI and blue dye remain effective and widely used in SLNB, limitations such as variable identification rates and the need for complementary techniques have led to interest in newer adjuncts such as indocyanine green fluorescence (ICG-F)^8,12-14,15^. The ICG-F method has been shown to be safe for early breast cancer and SLNB after NAC and can provide high levels of SLN detection comparable to the RI method^14-18^. In this study, we aimed to examine the effectiveness of adding ICG-F (triple method) to the dual method (DM) (RI+Isosulfanblue) for SLNB after NAC on SLN identification.

## METHODS

### Ethical approval

This study was approved by the Lütfi Kırdar City Hospital Ethics Committee (Date: 11/11/2020; approval number: 514/189/5). This study was conducted in accordance with the ethical standards defined by the Institutional Research Committee and the 1964 Helsinki Declaration. Informed consent was obtained from all patients. This research did not receive any specific grants from funding agencies in the public, commercial, or not-for-profit sectors.

### Study design

A total of 101 breast cancer patients scheduled for surgery after NAC at the General Surgery Clinic of Istanbul Kartal Dr. Lütfi Kırdar City Hospital were included in this study. Patients were allocated to groups by a manual lottery (drawing lots) method, ensuring that each patient had an equal chance of being assigned to each group. DM, including ICG (group 1) or DM alone (group 2), was used for SLN mapping.

### Inclusion and exclusion criteria

Female patients aged 18 years and over who completed NAC and had clinical stage T0-4, N0-3, or M0 primary invasive breast cancer according to the American Joint Committee on Cancer staging system were included in the study. Breast cancer patients who had previously undergone breast or axillary surgery, M1 stage patients and patients who could not be followed up were excluded from the study.

### Preparation of dual method agents for sentinel lymph node mapping

On the morning of the surgery day, patients were randomized to the DM or triple method group. In the DM, patients were referred to the nuclear medicine unit for Lymphoscintigraphy. 1 mL/111 MB pertechnetate (Tc-99m, TcO4-)/NaCl and 1 mg reduced human serum albumin were mixed and then incubated for 10 min at room temperature. Patients were checked with lymphoscintigraphy 30 min after the subareolar injection, and surgery was planned within 6 h after the injection. During the surgery, approximately 3 mL of isosulfanblue was injected into the patient from the subareolar area and massaged for 10 min.

### Preparation of indocyanine green and blue dye

**Figure 1:**
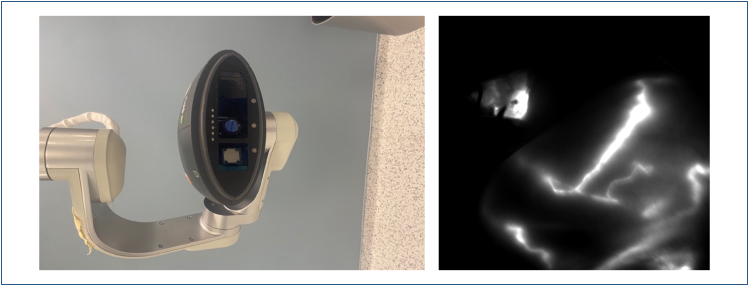
SPY 2001 Imaging System, (Novadaq, Bonita Springs, Florida) and transcutaneous progression and axillary involvement.

A volume of 1 mL methylene Blue dye (10%) was injected peritumorally in all four quadrants (total of 4 mL), 20 min prior to surgery. In the patients in the group in which indocyanine green (ICG) was added in addition to the DM, 0.6 mg ICG was dissolved in 10 mL of injectable distilled water. Notably, 3 mL of the prepared ICG dye was injected in the retro-areolar region for all patients irrespective of tumor location after the patient was intubated and massaged for approximately 10 min. Transcutaneous lymphatic progression was recorded. The SPY 2001 Imaging System (Novadaq, Bonita Springs, Florida), a device with integrated infrared, was used as the ICG-F imaging system (Figure 1).

### Sentinel lymph node identification

To clarify the timeline of the techniques, patients who were to undergo lymphoscintigraphy with Tc-99 were sent to the nuclear medicine unit on the morning of the operation. ICG and blue dye procedures were performed sequentially on the operating table before the operation. ICG was used in conjunction with infrared imaging and other techniques during SLNB.

A lymph node was considered to be an SLN when a fluorescent lymph node was observed by infrared imaging or a radioactive lymph node, including the warmest lymph node detected with the gamma probe and nodes showing more than 10% of the counted maximum. When malignancy was detected in the SLN by Frozen, axillary lymph node dissection (ALND) was performed. All surgical procedures were performed by an experienced breast surgery team.

### Outcomes

As the primary outcome, we compared SLN identification rates between the two groups. As a secondary outcome, we evaluated the number of SLNs. Additionally, the patients’ breast and axilla surgery, hormonal status, stage, response to treatment after NAC, SLNB definition, and SLN numbers were recorded and compared between both groups.

### Statistical analysis

The sample size was calculated for a superiority design, assuming an SLN identification rate of 80% for the DM group in the initially node-positive breast cancer patients with NAC and the SLN identification rate of 95% in the DM containing indosyanin green (ICG) group. To detect a 15% difference in the identification rate, this study required 58 patients, with a one-sided type 1 error rate of 10% and a power of 81%. Mean, standard deviation, median, lowest, highest, frequency, and ratio values were used in the descriptive statistics of the data. The distribution of variables is measured by the Kolmogorov-Smirnov test. The Mann-Whitney U test was used in the analysis of quantitative independent data. Chi-Square test in the analysis of qualitative independent data and Chi-Square test conditions were not met, the Fisher test was used. Comparison of the distribution between the DM and DM include in ICG groups was performed using the Chi-square test, Fisher's exact test, and the t-test. The remaining results were considered significant at a two-sided p-value lower than 0.05. The time to detection of the SLN was defined as the time from the skin incision to the extraction of the first SLN. The SPSS 28.0 program was used in the analyses.

## RESULTS

A total of 101 patients were included in the study, 45 in the DM group and 56 in the triple method group. Age range, min: 32–max: 75. Mean age was 56 (53.7±10.2) years. Although clinical T and clinical N stages were proportionally higher in the DM group between the groups, no statistically significant difference was found (p>0.05). The pathological complete response (PCR) rate was significantly (p<0.05) higher in the group using the DM. No significant difference was found in progesteron receptor (PR) and estrogen receptor (ER) positivity, cERB2 positivity, and KI-67 value between both groups (p>0.05). The biopsy rate taken from the preoperative suspicious ALN was also similar between the groups (p>0.05). The rates of breast-conserving surgery after NAC were 34.8 and 41.8%, respectively (p=0.47). ALND was applied to 65 patients, 67.4% in the DM group and 61.8% in the triple method group. Clinicopathological features and comparison between groups are summarized in Table 1, and SLN identification rates are shown in Table 2.

**Table 1:** Clinicopathological features and comparison between groups.

			Dual method				Triple method				P
			Average .±ss/n-%			Median	Average .±ss/n-%			Median	
Age			53.6	±	9.5	56.0	53.8	±	10.8	56.0	951^m^
Diagnosis		IDC	46		100%		52		94.5%		249χ^2^
		DCIS	0		0.0%		3		5.5%		
**Breast surgery**											
Mastectomy			30		65.2%		32		58.2%		470χ^2^
BCS			16		34.8%		23		41.8%		
**Axilla surgery**											
SLNB			15		32.6%		21		38.2%		560χ^2^
SLNB+ALND			31		67.4%		34		61.8%		
Clinic T	I		28		60.9%		27		49.1%		123χ^2^
	II		18		39.1%		24		43.6%		
	III		0		0.0%		3		5.5%		
	IV		0		0.0%		1		1.8%		
Clinic N	(-)		30		65.2%		27		49.1%		104χ^2^
	I		16		34.8%		14		25.5%		
	II		0		0.0%		12		21.8%		
	III		0		0.0%		2		3.6%		
pCR	Partial		22		47.8%		37		67.3%		42χ^2^
	Full		18		39.1%		12		21.8%		
ER	Negative		26		56.5%		27		49.1%		456χ^2^
	Positive		20		43.5%		28		50.9%		
PR	Negative		32		69.6%		33		60.0%		318χ^2^
	Positive		14		30.4%		22		40.0%		
HER2 NEU	Negative		14		30.4%		19		34.5%		661χ^2^
	Positive		32		69.6%		36		65.5%		
Ki-67			28.7	±	19.4	25.0	32.5	±	24.5	20.0	621^m^
Axilla biopsy		(-)	22		47.8%		31		56.4%		392χ^2^
		(+)	24		52.2%		24		43.6%		

χ^2^: Chi-Square test; ^m^Mann-Whitney U test. SLNB: sentinel lymph node biopsy; ALND: axillary lymph node dissection; pCR: pathological complete response; PR: progesteron receptor; ER: estrogen receptor. The bold values indicate statistically significant results (p<0.05), and the italic values represent mean±standard deviation.

**Table 2:** Sentinel lymph node number and identification rates, transcutaneous transmission.

	Dual method	Triple method	P
SLN identification rate	46/40 (86.9%)	56/53 (94.6%)	0.312
SLN identification rate of 2 SLN's and above	46/38 (82.6%)	56/50 (89.2%)	0.492
	Total study group		
	Min–max	Median	Aver.±ss/n-%
Total Number of SLNBs	2.0–10.0	4	3.9±1.4
Total Time of SLNBs	9.0–25.0	14.0	14.6±4.7
	(+)	(-)	
Transcutaneous Transmission (%)		38/56 (69.1%)	

SLN: sentinel lymph node; SLNB: sentinel lymph node biopsy.

The SLN identification rate was 86.9% in the DM group and 94.6% in the triple method group. The detection rates of two or more SLNs were 82.6% in the dual group and 89.2% in the triple group. There was no statistically significant difference between both the groups (p>0.05). In the study group, the average number of identified SLNs per patient was 4 (min: 2/max: 10). No significant difference was found between the time to find the first SLN and the total SLN between the two groups. Transcutaneous transmission was evaluated to demonstrate lymphatic progression in all patients in whom ICG was used. This progression was demonstrated in 38 of 46 patients (69.1%) in the triple method group.

When we examined the rates of identifying any sentinel lymph node (1st, 2nd, or other SLN) among the ICG, RI, and isosulfan blue methods in the triple method group, ICG alone was found to be significantly more successful than the other methods (p<0.005) (Table 3).

**Table 3:** Sentinel lymph node identification rates in the triple method of indocyanine green, radioisotope, and isosulfan blue.

	Indocyanine green (ICG)	Radioisotope (RI)	Isosulfan blue	
SLN identification rate	n: 51, 92.7%	n: 45, 81.8%	n: 35, 63.6%	p<0.05

SLN: sentinel lymph node.

No allergy, necrosis, or any side effects were observed in any of the patients due to preoperative procedures performed for SLN mapping.

## DISCUSSION

Following neoadjuvant chemotherapy (NAC), the optimal surgical approach remains a subject of debate; however, recent years have witnessed an increased inclination toward conservative strategies, particularly breast-conserving surgery^19^. The most important problem in evaluating the axilla after NAC is the identification and number of SLNs. FNR is also an important topic of discussion in this patient group. In the ACOZOG Z1071 study, the identification rate was 92.7% and FNR was 12.6%. But, when the DM was used, the FNR was 8.6%, and when there were three or more SLNs, the FNR was 9.1%^3^. In addition, the GANEA study including clinically node-negative and node-positive patients before NAC, identification rates have been reported as 94.6 and 81.5%, respectively, and FNRs as 9.4 and 15%, respectively^19^. In the SENTINA study, the identification rate of 99.1% in patients with SLNB before NAC and 80.1% in patients with clinically node-negative breast cancer after NAC was found. In the study in question, FNR was found to be 14.2%^9^. Although these large-scale studies have shown that surgeons cannot reliably detect all ALN metastases in patients with cN1 breast cancer after chemotherapy with SLN procedures, they have identified important factors that affect the likelihood of false-negative SLN. FNR was significantly lower when dual-agent mapping was used. In our study, we did not calculate FNR by performing axillary dissection in every patient. However, we investigated the effect of the triple method on the SLN detection rate and the role of ICG. To our knowledge, our study is the first to compare SLN identification rates between the triple method, including ICG-F and the DM, in breast cancer patients receiving NAC. The results showed that the SLN identification rate and the number of SLNs identified using the triplet method were similar to the DM. Additionally, the identification rate for ICG alone in the triple method was higher than for RI alone and isosulfan blue alone, although the difference was not statistically significant. However, in our study, no research was conducted on which patient subgroups ICG was more effective in.

The use of ICG-F for SLN mapping after NAC has been examined in previous studies^9,15,20,21,22^. In Chirappapha and colleagues’ study, when ICG was used with isosulfan blue, the SLN identification rate was 96.71% and FNR was 10% and this result was better than blue dye+isotope (p=0.041). When each method was examined separately, the SLN identification rate was found to be 93.22% with ICG, 81.78% with blue dye, and 53.87% with RI. When all three methods were used together, the identification rate was found to be 97.03%^23^. In a previous study evaluating the use of ICG-F, ICG-F was significantly better than blue dye, while SLN identification was similar to RI (Blue dye: OR 18.37; 95% GA, 8.63–39.10/RI: OR 0.81; 95% GA, 0.03–24.29). As a result, they stated that SLNs were detected in all clinically node-negative cases, and the identification rate in clinically node-positive cases was 93.8%^24^. Jung et al.'s study comparing SLN identification rates between ICG-F with RI (DM) and RI alone in breast cancer patients with NAC also demonstrates the feasibility of ICG-F. The SLN identification rate in the DM group was found to be higher than the RI-only group (98.3 vs. 94.7%) and the identification rate of ICG-F alone in the DM group^14^. In another study, the identification rate with dual dye was 94%, while it was 96% with ICG alone. The SLNB sensitivity rate and FNR were 97.6% versus 93.2 and 3.1% versus 6.2% in the combination of ICG and dual dye, respectively, the study found that ICG can be used reliably for SLNB in early breast cancer instead of dual dye^15^. The findings and the results in our study were found to be compatible with the literature. While the triple method trend appears better in detecting two or more SLNs, the difference was not statistically significant. The sample size may be insufficient to detect smaller but clinically meaningful differences. Even though the increase in SLN detection was not statistically significant, this finding could still have clinical relevance, particularly in improving the detection rates in challenging cases where additional confirmation might be needed. Transcutaneous transmission was demonstrated in 69.1% of patients. There was no statistically significant difference between the time to find the first and total SLN. In our study, ICG was found to be statistically more successful when the methods alone were evaluated in the triple group. The triple method was proportionally more successful in the SLN identification rate. Although these results are promising for the use of ICG and the triple method, they do not reveal clear clinical results. However, these statistics may change in studies to be conducted with larger sample groups and specific subgroups.

This study has some limitations. Although the SLN identification rate was higher in the triple method than in the DM, we did not find a statistically significant difference. This may be because the sample size of the study was too small to evaluate the power of the methods. Before NAC, we could not perform ALN biopsy in all patients and evaluate clinical lymph node metastasis using imaging methods. Another limitation of our study is the absence of oncologic outcomes such as axillary recurrence, disease-free survival, and overall survival (OS). Additionally, since ALND was determined based on SLN results, we did not calculate the FNR of SLNs. There are important studies in the literature showing that the FNR of ICG is acceptably low^14,15^. The aim of this study was to investigate the optimal method for SLN identification rate after NAC in breast cancer patients.

## CONCLUSION

We believe that the triple method will provide a safe effect on SLN biopsy. Furthermore, ICG-based SLNB detection will bring a significant change in the management of patients in centers where there is no access to a nuclear medicine department. Nevertheless, multicenter studies with larger patient samples are needed to confirm our results.

## Data Availability

The datasets generated and/or analyzed during the current study are available from the corresponding author upon reasonable request.
